# Effects of authentic leadership on the experiences of burnout and Turnover Intention among healthcare professionals

**DOI:** 10.12669/pjms.42.1.12686

**Published:** 2026-01

**Authors:** Badr K. Aldhmadi, Fahad D. Algahtani, Bilesha Perera

**Affiliations:** 1 Badr K. Aldhmadi, Ph.D., Department of Health Management, College of Public Health and Health Informatics, University of Ha’il, Saudi Arabia; 2 Fahad D. Algahtani, Ph.D. Department of Public Health, College of Public Health and Health Informatics, University of Ha’il, Saudi Arabia; 3 Bilesha Perera, MSc., Ph.D. Faculty of Medicine, University of Ruhuna, Galle, Sri Lanka., Department of Health Management, College of Public Health and Health Informatics, University of Ha’il, Saudi Arabia

**Keywords:** Authentic Leadership, Burnout, Healthcare professionals, Saudi Arabia, Turnover intention

## Abstract

**Objective:**

To consider empirically how authentic leadership (AL) effects burnout experience (BE) and the employee turnover intention (TI) in healthcare settings.

**Methodology:**

This study followed a cross-sectional design. Furthermore, a close-ended, online survey was conducted between March to May, 2025. It concludes with the data collection from 318 healthcare professionals. The participants mainly include doctors, and nurses at leadings hospitals in Ha’il region of Kingdom of Saudi Arabia (KSA). Finally, the analysis of data includes descriptive statistics, reliability analysis, correlation matrices, regression analysis and diagnostic tests. All of these analyses were performed using SPSS 27 version.

**Results:**

The descriptive summary volunteer participants showed that majority of them are predominantly female (e.g., nurses). Most of these participants were aged between 31-40 years and holding bachelor’s qualifications. Moreover, empirical results from regression analysis revealed some interesting relationships. For example, the results indicate that AL is positively linked with BE (β = 0.0374, *p* = 0.006). However, a negative impact was also found for TI (β = −0.2805, *p* < 0.001). The model was statistically fit and provide a reasonable explained variations for BE (*R*^2^ = 0.0241, *F* = 7.81, *p* = 0.006). and TI (*R*^2^ = 0.1035, *F* = 36.51, *p* < 0.001).

**Conclusion:**

The study concludes a strong positive impact of AL on BE while a strong negative impact of AL with TI. These results suggested a strong effort from the leadership is necessary to enhance the retention and wellbeing of employees in healthcare setting. Finally, studies can be made to further examine the cultural and organizational factors that may moderate these impacts.

## INTRODUCTION

The leadership has an important effect on workforce experiences and institutional outcomes in modern healthcare systems. The existing literature indicated that AL provides favorable outcomes within healthcare setting. It is an approach to increase self-awareness, relational transparency, balanced information processing, and a strong internal moral framework.^1^ Apparently, these abilities present an environment of trust and psychological safety healthcare professionals. Consequently, AL impacts employee engagement, psychological well-being, and resilience in different contexts and regions.^2^ This leadership style goes a long way in ensuring supportive and effective workplace in healthcare setting

BE is a multidimensional psychological condition. It ensures chronic occupational stressors, that has reached epidemic levels in the global healthcare sector.^3^,^4^ The latest literature provide empirical evidence for doctors, nurses, and allied health professionals about their emotional exhaustion, reification, and reduced personal achievement.^5^,^6^ These findings have indicated that burnout is pervasive across multiple disciplines and healthcare context. The adverse impacts of BE are wide-ranging. It causes to decrease the well-being of healthcare professionals. It also reduces the quality of patient’s care. As a result, it impaired organizational performance.^7^ Existing latest studies also indicate that increasing burnout causes to increase medical errors.^8^,^9^ Furthermore, it reduces patient satisfaction, and compromised patient safety. These relationships accentuate the direct effects of burnout on healthcare performance. Most importantly, BE strongly affects healthcare professionals’ intentions to leave their positions.^10^ In this way, employee turnover intensifies workforce shortages and imposes substantial financial burdens on health systems. Additionally, costs associated with employee turnover include costs related to recruitment, training, and productivity loss. The continuous replacement of experienced personnel can disrupt team functioning and continuity of care. The empirical evidence from some latest research studies indicated that BE and TI is associated with increased workload pressures.^11^,^12^ This leads to a self-reinforcing cycle of strain and attrition. Consequently, addressing BE is vital to sustain healthcare workforce. It also helps to maintain a quality service in healthcare setting.

The existing literature highlighted the increasing impact of leaderships styles by decreasing BE, and improving employee retention in healthcare.^13^ Leaders create an organizational climate with stress reduction, and promoting wellbeing of workforce.^14^ A number of empirical studies have focused primarily on nurses, and physicians along with allied health staff, and admins.^15^ However, the existing literature still lacks empirical evidence for the association between AL and TI in across diverse roles of workforce in healthcare settings. Furthermore, institutional contexts, and their cultures might vary in terms of how leadership influences BE and TI.^16^ Hence, fresh research is required on diverse healthcare settings. In this way, the study intends to fill these gaps by investigating the impact of EL on BE and TI healthcare professionals with diverse departmental belonging. Furthermore, the results can help leadership development programs and organizational policies that promote the well-being and job satisfaction of employees. Finally, this study contributes empirical evidence supporting effective sustainable management of the healthcare workforce.

## METHODOLOGY

The study followed cross-sectional design. The respondents include healthcare professionals (e.g., nurses and doctors) from Ha’il region of KSA. These participants were serving different specialties and department within healthcare. A total 350 (including nurses and doctors) healthcare professionals were targeted for data collection. However, 318 of them responded to the online questionnaire. It considered 90% prevalence of adequate understanding of AL with a 5% margin of error, and 95% level of confidence. The data was collected during March-May 2025.

### Ethical considerations:

The research and ethics committee from University of Ha’il provided ethical approval (No.: H-2021-152, dated: May 3, 2021) for conducting this study.

### Informed consent:

Before formal data collection, the participants were informed about research aims, their rights, and how we maintain their confidentiality. The online survey started only after they confirmed their voluntary participation.

### Inclusion and Exclusion criteria:

Participants, those who agreed to voluntarily participate in this study were included and those who declined were excluded from this research.

### Instrument and Variables:

Information was collected using a structured, validated self-administered questionnaire consisting of four main sections. First, demographic and professional information such as sex, age, marital status, educational qualification, medical specialization, years of professional experience, hospital affiliation and department of work were obtained. This section was designed to describe the characteristics of the sample and to allow subgroup analyses. The second section measured authentic leadership perception using the Authentic Leadership Questionnaire (ALQ Version 1 Rater), which consists of 16 items that assess how frequently participants perceive leadership behaviors consistent with the description provided by authentic leadership theory. These behaviors include self-awareness, relational transparency, ethical decision-making and balanced processing of information. Each item was responded to by the participants on a 5-point Likert scale, from 0 (“Not at all”) to 4 (“Frequently, if not always”). This allows for the measurement of the perception of authentic leadership. The scale was adapted from recent research by Mao, Kang.^17^

The third section was the TIS, a 15-item scale used to capture the multifaceted nature of employees’ thoughts, feelings and behaviors pertaining to their intention to quit their current organization. Items correspond to factors including general job dissatisfaction, active job searching and emotional responses to the work environment. Responses were measured on a 5-point scale from 1 (“Never”) to 5 (“Always”). The scale produced a reliable metric of turnover intention adapted from recent research by Kavaklı and Yildirim.^18^

The fourth part measured burnout using the Oldenburg Burnout Inventory (OLBI). it comprises of 16 statements examining two of its dimensions (e.g., exhaustion, and disengagement). Several items are reversely keyed to maintain response consistency. Because of its sound psychometric properties, OLBI is acceptable in measuring burnout among health professionals. The scale was adapted from a recent study by Yaowapak, Lortrakul.^19^ As the participants were employed in healthcare sector from KSA. Therefore, in order to confirm their complete understanding of each survey item, both version (e.g, English and Arabic) were distributed to volunteers.

### Sampling approach and Data collection:

In order to confirm maximum participation and representations from respondents, a convenience sampling approach was adopted. Google form was used to collect data. It is convenient and accessible for busy healthcare professionals.

### Data analysis:

The data analysis of this collected online data was performed using SPSS 27 version. It includes sample summary, reliability analysis, Pearson correlation, and regression analysis. Furthermore, some diagnostics tests like normality, multicollinearity, independence of errors, and heteroscedasticity were also performed.

## RESULTS

The summary of participant’s attributes like gender, profession, education, age, and their professional experience is shown in Table-I. It shows that 57.9% of participants were female, while 42.1% were male. The majority of respondents (71.1%) were nurses, with physicians constituting 28.9%. Most participants had bachelor’s (69.2%), while some of the had a master’s (16.4%) or doctorate (10.7%) qualifications. The age distribution was broad, with 26.4% aged 31-35 years and 18.9% aged 36-40 years. The largest proportion had between 6 to 10 years of professional experience (30.2%), while 27.0% had one to five years of experience. The table also indicated geographically that the majority of participants worked at King Salman Specialist Hospital in Hail (74.8%).

**Table-I:** Participant Summary.

Characteristics	Categories	N	%	Characteristics	Categories	N	%
Gender	Male	134	42.1%	Hospitals	Hail General Hospital, Hail	32	10.1%
	Female	184	57.9%		King Khalid Hospital, Hail	40	12.6%
Marital	Single	116	36.5%		King Salman Specialist Hospital, Hail	238	74.8%
	Married	202	63.5%		Maternity and Children Hospital in Hail	8	2.5%
Age	20-25	50	15.7%	Profession	Nurse	226	71.1%
	26-30	54	17.0%		Physician	92	28.9%
	31-35	84	26.4%	Departments	Anesthesia	6	1.9%
	36-40	60	18.9%		Burn unit	8	2.5%
	41-45	24	7.5%		Cardiac	118	37.1%
	46-50	24	7.5%		Emergency	58	18.2%
	51 or above	22	6.9%		Endocrinology	2	0.6%
Education	Diploma	12	3.8%		ENT	2	0.6%
	Bachelor	220	69.2%		Intensive Care Unit	18	5.7%
	Master	52	16.4%		Medical	26	8.2%
	Doctorate	34	10.7%		Nephrology	4	1.3%
Nationality	Egyptian	48	15.1%		Neurology	2	0.6%
	Indian	36	11.3%		Obs and Gynae	2	0.6%
	Jordon	2	0.6%		Oncology	4	1.3%
	Pakistani	4	1.3%		OPD	14	4.4%
	Philippines	72	22.6%		Orthopedic	10	3.1%
	Saudi	130	40.9%		Pediatrics	4	1.3%
	Sudan	12	3.8%		Psychiatry	2	0.6%
	Syrian	10	3.1%		Pulmonology	2	0.6%
	Yaman	4	1.3%		Radiology	18	5.7%
Experience	< 1 year	26	8.2%		Surgical	14	4.4%
	1-5 years	86	27.0%		Urology	4	1.3%
	6-10 years	96	30.2%				
	11-15 years	62	19.5%				
	16-20 years	20	6.3%				
	> 20 years	28	8.8%				

The variables of this study are listed in Tale-II. It indicates mean and standard deviations for these measures. For example, the table indicated that AL has a mean score of 2.39. It indicates healthcare professionals have moderate perception about AL. Similarly, TI indicated a mean score of 2.87, while BE represented a mean of 2.18. Table-II also indicated the internal validity and reliability of these measures in the form of Cronbach Alpha (CA). It indicated a CA value 0.961 for AL, 0.895 for TI, and 0.736 for BE. These CA values meet the threshold of internal reliability and validity (CA > 0.70). Moreover, Table-II also indicated the Pearson correlation between these variables to understand the possible associations and direction between them. For example, the table shows a negative significant association between AL and TI with a coefficient of -0.322, having a p-value less than 0.01. Additionally, the same table indicated a positive significant association between AL and BE with a coefficient of 0.155, and having a p-value less than 0.01. However, the correlation analysis didn’t confirm any significant association between TI and BE.

**Table-II:** Reliability, Descriptive and Correlation Analysis.

Var	Items	α	N	Min	Max	Mean	Std. Dev	AL	TI	BI
AL	16	0.961	318	0.00	4.00	2.39	0.96	1	-0.322**	0.155**
TI	15	0.895	318	1.00	5.00	2.87	0.84	-0.322**	1	-0.021
BE	16	0.736	318	1.00	4.00	2.18	0.23	0.155**	-0.021	1

** Correlation is significant at the 0.01 level (2-tailed).

Var = Variable name, Items = Number of items in a variable, α = Cronbach alpha, N = number of respondents, Std. Dev = Standard Deviation, AL = Authentic Leadership, TI = Turnover Intention, BE = Burnout Experience.

Diagnostics test for confirming the model’s suitability based on validating assumptions is shown in Table-III. At first, the DW statistics reported a value of 1.944 for BE, and 1.816 for TI. As these values are close to 2 (meeting the threshold), therefore, independence of residuals was confirmed. Additionally, a VIF values of 1.000 (should be less than 5) confirmed that there is no multicollinearity between independent variables

**Table-III:** Diagnostic Tests.

Assumption	Test	DV = BE		DV = TI		Decision
		Test Statistics	P-Value	Test Statistics	P-Value	
Error’s Independence	DW	1.944	-	1.816	-	Residuals are Independent
Multicollinearity	VIF	1.0000	-	1.0000		Multicollinearity is absent

DW = Durbin Watson, VIF = Variance Inflation Factors.

The regression analysis to investigate the impact of AL on BE, and TI are shown in Table-IV. The analysis in the table showed that AL strongly enhances BE (β= 0.0374, *p* = 0.006). It suggested that a higher AL is associated with greater burnout. However, the same table showed that AL strongly decreased TI (β= -0.2805, *p* < 0.001). It suggested that stronger AL perceptions correspond with lower TIs. The model explained 2.41% of variance in BE (*R^2^ = 0.0241, F = 7.81, p = 0.006*) and 10.35% of variance in TI (*R^2^ = 0.1035, F = 36.51, p < 0.001*). It demonstrated a strong and overall fit of this model.

**Table-IV:** Regression Analysis.

	DV = BE				DV = TI			
Effects	β	ST. Err	t-value	p-value	β	ST. Err	t-value	p-value
AL	0.0374	0.0134	2.79	0.006	-0.2805	0.0464	-6.042	0.000
Constant	2.0867	0.0345	60.50	0.000	3.5425	0.1195	29.63	0.000
R-Square	0.0241				0.1035			
R-square adjusted	0.0210				0.1007			
F-Value	7.8114				36.5088			
Model Fit (P-value)	0.0056				0.0000			

## DISCUSSION

This research concludes how AL, BE, and TI are related within healthcare setting of KSA. The finding inferred that AL has a small but significant positive impact on burnout, which is contrary to existing studies on such relationships.^20^ Such unexpected findings create a unique pressure for the healthcare context of Ha’il region in KSA. In some cases, transparency and ethical standards may be a pre-requisite for authentical leaders which may cause work-related stress among staff.^21^ Such requirements in the presence of supportive leadership may damage the subordinates emotionally.^22^ The conclusive results also indicate a complex connection between leadership styles and staff wellbeing. Specifically, it is noteworthy that the subordinates with authentic leadership may show higher burnout and lower turnover intention. Hence, AL may enhance commitment and attachment to the organization. This effect appears even when employees are exposed to considerable emotional strain. This pattern adds a novel perspective to the AL literature. It indicates that authentic leadership alone may not sufficiently buffer burnout in high demand healthcare environments. However, the findings indicates that AL plays a negative role in impacting the TI. This result matches with the argument of most studies that indicates AL as a motivator for employee retention, job satisfaction and establishing trust.^23^,^24^ Therefore, the study contributes to the literature by investigating these relationships in KSA’s healthcare context. It is underrepresented in leadership research.^25^,^26^ These findings emphasize the importance of AL in decreasing TI. It is a critical issue given workforce shortage locally and globally.^27^,^28^ From a clinical and organizational perspective, these results underscore the potential of authentic leadership development as a practical strategy. This method fosters stabilization among the workforce of healthcare in order to sustain care without any dis-continuity. It may also reduce negative downstream effects of high turnover on patient safety, quality of care, and organizational costs. The conclusive results can guide the retention policies, succession planning and leadership training in healthcare settings from similar context.

The positive impact of AL and burnout may call for urgent attention towards organizational and contextual factors that may change these relationships.^29^-^31^ Factors such as workload intensity, resource availability and cultural norms may alter how AL impacts burnout across different settings.^21^,^32^ Additionally, role ambiguity and emotional labor inherent in healthcare work may further complicate this association. Future research should examine these moderating variables in greater detail.^15^,^33^ Specifically, subsequent studies should investigate how organizational and cultural moderators shape the influence of authentic leadership. These moderators include organizational culture and psychological safety. They may affect both burnout and turnover intention. A female dominance was observed in respondent summary having diverse education and experience levels. These characteristics did not significantly moderate the relationships between AL, burnout and turnover intention. This aligns with previous research indicating that demographic variables often have limited influence on leadership outcomes.^25^,^34^-^36^ Nonetheless, future studies should explore additional factors such as organizational culture and psychological safety, which may shape these dynamics. By shifting the focus from individual demographic characteristics to organizational and cultural conditions, future research can better identify leverage points. These influencing factors can guide the interventions to improve the retention and wellbeing of staff.

Finally, the study makes several significant contributions for existing literature in leadership and healthcare. First, it provides empirical evidence from an underrepresented healthcare context in KSA. In doing so, it addresses a geographical gap in authentic leadership research. Second, it highlights a novel and seemingly paradoxical pattern in the effects of authentic leadership. Authentic leadership can coexist with higher burnout while still being associated with lower turnover intention. This suggests that healthcare professionals may remain committed to their organizations despite experiencing emotional exhaustion. Such commitment has important implications for the early identification and management of burnout. Third, the study underscores the need to consider both individual and organizational outcomes when evaluating leadership styles. Scholars should not assume that positive effects on one dimension, such as retention, automatically imply benefits for another. For instance, it is not necessary that increasing the retention may lead to wellbeing of employees psychologically.

Additionally, the results also confirm that AL by itself may not protect healthcare professionals from burnout in high pressure environments. Therefore, leadership development programs should be complemented by organizational interventions that address workload, staffing, resource allocation, and emotional support. For clinicians and managers, the results emphasize the importance of routinely monitoring burnout, even in well-led teams. These teams may be led by leaders who appear effective and supportive. The findings also highlight the need to implement targeted interventions, such as resilience training and peer support. It is important to consider some supplementary mechanisms of reducing adverse outcomes by improving psychological safety.

### Strengths of the study:

Focusing on the healthcare settings in a real world is first and foremost strength of this research study. This specific context of healthcare professionals indicates high demands, and shortages of workforce. Therefore, the findings are practically relevant for professionals as well as for healthcare organizations. The research considers a diverse population for studying the healthcare professional from KSA’s Ha’il region. This diversity of workforce not only provides dynamics of leadership but also unique context of regions.

### Limitations:

A number of limitations were found with this study. First limitation is the generalizability of the findings of this study. The study is restricted to healthcare professionals in Ha’il region of KSA only. Therefore, its findings are not generalized on other regions. Second important limitation is the self-reported measures of questionnaire items. It creates a potential bias in providing responses. The third limitation is cross-sectional research design of this study. It confines the underlying interpretation using causes and effects.

## CONCLUSION

The research concludes with empirical support of impacting turnover intentions through authentic leadership for healthcare professionals in Ha’il region in KSA. The authentic leadership helps to decrease the turnover intentions. However, the same shows low positive association with BE. The results indicate the significant role of leadership in reducing employee turnover intention. The findings suggested to include resource constraints and employee workload as contextual factors for understanding their impacts on burnout experience. Moreover, gender, profession, and experience level as personal demographic attributes of healthcare professionals showed no robust impacts. It indicates that leadership effect might be transcending these attributes. It is crucial for enhancing the stability and wellbeing of workforce in healthcare context. It is only possible by developing tactics for leadership and balancing the qualities of authentic leadership. Therefore, in order to improve the effectiveness of leadership in a diverse context of healthcare, further studies are needed.

### Recommendations

Future studies can use longitudinal research design and can consider multiple healthcare context. Moreover, future research can also consider some intervention models that may consider organizational changes, and leadership development as moderators. By using such moderators, future research can target psychological safety, support of resources, and employee workload. Finally, it is important to understand the subjective perceptions of healthcare professionals for examining the relationship between retention, burnout, and leadership. For this purpose, it is suggested to consider the qualitative aspects of this study.
